# Death Concerns, Benefit-Finding, and Well-Being During the COVID-19 Pandemic

**DOI:** 10.3389/fpsyg.2021.648609

**Published:** 2021-05-19

**Authors:** Cathy R. Cox, Julie A. Swets, Brian Gully, Jieming Xiao, Malia Yraguen

**Affiliations:** Department of Psychology, Texas Christian University, Fort Worth, TX, United States

**Keywords:** COVID-19, coronavirus, death, existential anxieties, benefit-finding, well-being

## Abstract

Because of the coronavirus (COVID-19) pandemic, reminders of death are particularly salient. Although much terror management theory research demonstrates that people engage in defensive tactics to manage mortality awareness, other work shows that existential concerns can motivate growth-oriented actions to improve health. The present study explored the associative link between coronavirus anxieties, fear of death, and participants' well-being. Results, using structural equation modeling, found that increased mortality concerns stemming from COVID-19 were associated with heightened benefit finding (e.g., relationship investment, gratefulness, patience) from the pandemic. Increased benefit finding, in turn, was related to higher life satisfaction, meaning in life, self-esteem, resilience, and vitality while also correlating negatively with depression and stress scores. There was no evidence for reverse mediation in that fear of mortality did not predict well-being through coronavirus worries. Overall, although many persons have experienced mental health concerns (e.g., fear, stress) as a function of the COVID-19 pandemic, our findings demonstrate positive benefits that paradoxically follow in terms of an increased appreciation of life, improved relationships, and better health.

## Death Concerns, Benefit-Finding, and Well-Being During the COVID-19 Pandemic

The 2019 coronavirus (COVID-19) pandemic has created a health and economic crisis in the United States and worldwide. According to the Centers for Disease Control and Prevention [Centers for Disease Control Prevention (CDC), 2021], over 31 million Americans have been diagnosed with the illness, with about 564,000 deaths stemming from it. To provide some context, COVID-19 has now claimed more lives than the number of U.S. soldiers killed in World War I (116,516) and World War II (405,399; Ducharme, 2020) combined. Not only has death become increasingly salient, but people's life meaning systems have been compromised considering rising unemployment, fluctuating stock prices, general economic chaos (e.g., product scarcity), and social isolation. While heightened mortality awareness can lead to greater negativity and defensiveness (e.g., Pyszczynski et al., 2015), increased fear and anxiety can also contribute to meaning and post-traumatic growth (e.g., Wong and Tomer, 2011; Schippers and Ziegler, 2019; De Jong et al., 2020). Building on this, the present study examined whether benefit finding (i.e., a positive life change; Carver and Antoni, 2004) associated with higher existential concerns from COVID is related to greater emotional and psychological well-being.

Before turning to the literature, it seems important to define what is meant by *meaning*. Meaning, broadly speaking, is the extent to which persons perceive their lives as being meaningful (Steger, 2018). Theorists and researchers have made distinctions between different types. For example, meaning can be global (e.g., generalized; Park, 2010), situational (i.e., how people make sense of tragedy and trauma in their lives; Park; Steger), ultimate (i.e., everyone has meaning, with the goal to discover and give it life; Frankl, 1959), and existential (i.e., having understanding, purpose, and a sense of significance; Reker and Wong, 1988; George and Park, 2014; Martela and Steger, 2016), just to name a few. For this paper, meaning will be about both existential and situational meaning. In other words, the extent to which individuals can create a meaningful reality (i.e., existential) in response to the fear and anxiety associated with the coronavirus pandemic (i.e., situational).

## Experimental Existential Psychology: Terror Management Theory (TMT)

Experimental existential psychology is a field focused on understanding how persons cope with the realities of the human condition. This includes a sense of self-awareness (i.e., how we think about ourselves), our relationship with others (e.g., isolation), the ability to create a meaningful and satisfying life, and the capacity to regulate anxieties associated with mortality awareness. For example, Rank (1936) argued that the human condition was characterized by both life and death concerns, with similar reasoning seen in Frankl (1959) work on meaning (i.e., choice). Other existential theorists include May (1953) writings on loneliness and anxiety, Fromm (1941) work on freedom pursuit and avoidance, and in the death-related theories of Becker (1973), Lifton (1979), and Yalom (1980). As humans, we are continuously reminded of our mortality – for example, in seeing a gruesome car accident, through social media (e.g., the news), and in experiencing the death of a loved one. Significant global events can also prime thoughts of death, including instances of war, terrorism, and disease (e.g., COVID-19, Ebola). Although the regularity in which individuals ponder their existence varies, there is much theoretical and empirical evidence to suggest that existential anxieties have significant influence on persons' thoughts, attitudes, and behavior, both consciously and unconsciously.

From the perspective of TMT (Pyszczynski et al., 2015), the awareness of death combined with the human desire for life (i.e., self-preservation) has the potential to create extreme anxiety or terror. People can defend themselves against mortality concerns either literally (e.g., belief in an afterlife; supportive partnerships) or symbolically (e.g., nationalistic pride; religion) through a tripartite defense system comprised of cultural beliefs (i.e., worldviews), self-esteem, and close relationships. Over 30 years of research within the social-psychological tradition has demonstrated that: (a) reminders of mortality increase belief striving, self-esteem maintenance, and relationship validation; (b) bolstering cultural worldviews, self-worth, and attachments reduce anxiety in response to threat; (c) attacking the integrity of the anxiety-buffering defense system leads to a heightened accessibility of death-related concerns; and (4) activating thoughts of one's cultural beliefs, close others, and boosts to self-esteem after mortality salience reduces death cognition and the need to engage in other terror management defenses. The three components of the psychological defense system have been found to be interchangeable (Hart et al., 2005), with meta-analytic results showing a bidirectional association between mortality awareness and increased belief striving (Burke et al., 2010), and alternatively, compromised worldviews resulting in greater death-thought accessibility (Steinman and Updegraff, 2015).

Most TMT work has focused on the extent to which individuals engage in heightened aggression and defensiveness (e.g., belief biases and distortions) when existential concerns are salient. Mortality induced participants have been shown to express increased liking for persons who support their cultural worldviews and heightened negativity toward those who do not. For instance, reminders of death (relative to control conditions) lead to greater feelings of greed and materialism (e.g., Kasser and Sheldon, 2000), promote stereotypic thinking and racism (e.g., Schimel et al., 1999), and support the derogation and annihilation of persons who harbor values and beliefs different from one's own (e.g., Pyszczynski et al., 2006). Individuals may also engage in health-compromising behaviors (e.g., smoking, tanning) following reminders of death if such activities validate cultural worldviews and self-esteem (Goldenberg and Arndt, 2008). These findings have collectively contributed to the notion that mortality awareness fosters hate and destruction (i.e., the “dark side” of human nature). As argued by others (e.g., Wong and Tomer, 2011), one of the limitations of TMT is its overemphasis on the negativity associated with death while ignoring life-enhancing and meaning-making strategies. This is consistent with Frankl's (1959; also see Yalom, 1980) view that people can flourish and grow, even (or especially) in the face of adversity, to achieve a meaningful existence.

## Death, Meaning, and Well-Being

Inherent in the TMT tradition is that people are trying to construct and maintain meaning when faced with mortality awareness. For example, research has found that persons reminded of death are likely to invest in their close relationships (e.g., Mikulincer et al., 2003), or religion (Vail et al., 2009), with both being significant contributors to a heightened sense of meaning. Routledge and Juhl (2010) directly found that individual differences in meaning in life moderated the effects of mortality salience on self-reported anxiety. In other words, participants reported a greater fear of death following reminders of mortality only if they scored low (rather than high) on life meaning. Not only does meaning provide a sense of psychological equanimity in the face of existential anxieties, but death concerns can lead to personal growth and expansion. Kosloff and Greenberg (2009) randomly assigned persons to a death or control condition and had them think about their ideal life with respect to growth-oriented (e.g., meaningful relationships, personal growth) or culturally derived goals (e.g., wealth, fame). When asked to assign 100 poker chips to different cards, mortality-induced individuals re-prioritized intrinsic life goals over extrinsic ones. These effects have been found longitudinally (e.g., Lykins et al., 2007; Heflick et al., 2011) and retrospectively following a natural disaster (i.e., an earthquake; Lykins et al.). This work collectively suggests that even if people engage in a defensive denial of their mortality, it is also possible for death concerns to have downstream consequences for living an authentic and meaningful existence (see e.g., Rogers et al., 2019 for a review).

Although there is evidence demonstrating that people seek meaning in response to mortality salience, there are a limited number of studies exploring growth-oriented defenses in response to a real-world catastrophe (i.e., COVID-19; e.g., Schippers and Ziegler, 2019; De Jong et al., 2020; Trzebiński et al., 2020). This is especially important, as research has found that uncertainty in everyday events can lead to trauma and loss (e.g., Updegraff et al., 2008). de Jong and colleagues, for example, have argued that the emotions experienced during the coronavirus pandemic are like the grief felt from losing a loved one. In other words, the sadness and emptiness related to the deterioration of normality (e.g., social distancing, isolation) experienced throughout the 2020 pandemic can contribute to meaninglessness and lower well-being (de Jong et al.). Research has found, more generally, that meaning presence (i.e., having aims, goals, and life direction) and significance (i.e., the inherent value of living a meaningful existence; Martela and Steger, 2016) are positively related to post-traumatic growth following bereavement (Sawyer and Brewster, 2019) and trauma (Updegraff et al., 2008). Regarding COVID-19, Trzebiński et al. (2020) showed that higher levels of life satisfaction and meaning resulted in lower levels of anxiety and emotional distress stemming from the pandemic. These studies thus collectively suggest that a life full of meaning, among other factors, may serve an anxiety-buffering function against fear and improve health. At the same time, a prolonged search for meaning may lead to increased negative outcomes and feelings of hopelessness (Updegraff et al., 2008; De Jong et al., 2020).

Additional research on meaning-making strategies has focused on the characteristic of benefit finding (BF). Benefit finding is when people look for positive aspects in challenging life situations (e.g., illness, trauma) to learn and grow from these experiences: “finding the silver lining” (e.g., Carver and Antoni, 2004). This might include increased feelings of social connectedness, acquiring a deeper understanding of the self, and reprioritizing life goals. According to Taylor (1983), perceiving benefits in stressful health domains (e.g., cancer, HIV) can be utilized to counteract disease negativity and help individuals create a meaningful reality, gain mastery, and maintain self-esteem in the face of serious illness. In this way, adaptation through BF may reduce stress and improve psychological and physical health. This is supported by evidence demonstrating that increased stress-related growth is associated with lower levels of depression, higher positive affect (e.g., happiness, hope, optimism, joy), and better well-being (e.g., higher immune functioning, reduced disease severity, longevity; see e.g., Bower et al., 2009 for a review). Although BF is generally measured as an individual difference regarding people's general disposition (Carver and Antoni; Tomich and Helgeson, 2004), it can also be manipulated through writing prompts (Mosley and Branscombe, 2020) or measured as a state-like construct.

## The Present Research

Integrating work on TMT, BF, and well-being, the purpose of the current study was three-fold. First, prior terror management research has found a positive association between disease salience (e.g., cancer; Arndt et al., 2007; Ebola; Arrowood et al., 2017) and mortality-related concerns. To the extent that the coronavirus pandemic leaves people feeling existentially vulnerable, it was hypothesized that participants' heightened fear of COVID (FOC) would be positively associated with greater concerns about death (Hypothesis 1).

Second, to the extent that existential worries have the potential to increase meaning-making strategies (Sawyer and Brewster, 2019; De Jong et al., 2020; Trzebiński et al., 2020), with BF emerging as one factor from such negative experiences (e.g., Carver and Antoni, 2004), we believed that individuals would engage in increased stress-related growth (i.e., BF) from the association between heightened death awareness and coronavirus anxieties (Hypothesis 2). This would be consistent with general work arguing that optimal functioning depends on people's ability to transform the negative and strengthen the positive when existential concerns are salient (e.g., Wong and Tomer, 2011). With respect to TMT specifically, people have been found to pursue a life of meaning (e.g., authenticity, intrinsic goal pursuits) when death concerns are salient (see e.g., Rogers et al., 2019).

Finally, a structural equation model was constructed to test the connection between COVID fears, mortality salience, BF, and well-being outcomes (i.e., life satisfaction, meaning in life, self-esteem, resilience, vitality, depression, and stress). There is a large body of work demonstrating that meaning in life, including BF, is an important aspect of well-being. For instance, a life perceived as being more meaningful is associated with reduced anxiety and depression (e.g., Zika and Chamberlain, 1992), greater emotional and psychological health (e.g., Zika and Chamberlain, 1987), heightened physical functioning and reduced illness severity (e.g., Steger, 2018), a drop in post-traumatic stress symptoms among military veterans (Owens et al., 2009), and a longer life expectancy (Hill and Turiano, 2014). Similar effects have also been found with BF (e.g., Helgeson et al., 2006; Bower et al., 2009). Given that prior research has found better health stemming from increased meaning seeking and post-traumatic growth, it was hypothesized that greater BF related to coronavirus and death-related concerns would be correlated with higher emotional and psychological well-being (Hypothesis 3).

## Method

### Participants

Our initial sample was comprised of 451 individuals from two populations: (a) undergraduate students taking introductory classes who participated in exchange for partial course credit (*n* = 129) and (b) workers recruited from Amazon's Mechanical Turk (MTurk) who were compensated $1.00 for their survey completion (*n* = 322).^1^ Additional restrictions for MTurk persons included being in the United States, having a task approval rate of at least 90%, and taking part ≤ 100 prior HITs (i.e., human intelligence tasks). Upon closer inspection, 68 responses were from the same MTurk worker ID (although the Qualtrics Ballot Box Stuffing feature was activated), 33 persons failed to pass bot detection tasks (e.g., Captcha), 25 individuals reported being careless (i.e., “Did you read the questions carefully and answer them honestly?” “In your honest opinion, should we use your data in our analysis of the study?”), 83 participants failed attention checks within the survey (e.g., “Please select “strongly agree” in response to this question”), and four people had missing data. The final sample was comprised of 238 participants (students = 116 [a 10% reduction], MTurk workers = 122 [a 62% reduction] – see Discussion for further comments). Individuals were between 17 and 71 years of age (*M* = 25.03, *SD* = 8.68), predominantly female (67%), mostly Caucasian (69%; 9% Asian, 10% Hispanic, 7% Black/African American, 5% Other), and were equitable in relationship status (i.e., 53% in a relationship, 47% single).

### Materials and Procedure

Upon receiving Intuitional Review Board (IRB) approval, a Qualtrics survey on “personality and attitudes” was posted on sona-systems.com (i.e., student participants) and MTurk for the month of April (i.e., 4/1/20-4/28/20). The data reported here was part of a larger study on the association between COVID-19 concerns and well-being (see Swets and Cox, 2021 for additional information).^2^ Of the following scales, FOC and death concerns were counterbalanced to eliminate any order effects. Additionally, all well-being measures were presented in random order. The study took ~15–20 min to complete, after which participants were thanked and debriefed (i.e., additional study information provided).

#### FOC

Following some general personality measures (i.e., aloneliness; Coplan et al., 2019; nostalgia proneness; Barrett et al., 2010; the Ten-Item Personality Inventory [TIPI; Big 5]; Gosling et al., 2003), participants answered three items developed for the purpose of the present study. They included: “I am really scared of getting sick from the coronavirus,” “The topic of the coronavirus troubles me greatly,” and “The coronavirus holds nothing for me to fear” (reverse-scored). Participants were asked to respond based on how they felt “right now.” Items were measured on a 7-point Likert scale (1 = *strongly disagree*; 7 = *strongly agree*; α = 0.67).^3^

#### Fear of Death (FOD)

Following Cox et al. (2012), three items assessed people's present concerns about mortality: “I worry about the fragility of life,” “I think often about how short life really is,” and “The thought of my mortality bothers me.” Responses were made on a 7-point Likert-type scale (1 = *strongly disagree*; 7 = *strongly agree*; α = 0.78).

#### BF

To measure growth stemming from the COVID-19 pandemic, participants were asked to complete Tomich and Helgeson (2004) 14-item BF measure. Items were reported on a 7-point scale (1 = *strongly disagree*, 7 = *strongly agree*) with statements reflecting the beneficial effects of the coronavirus pandemic in association with close relationships (e.g., “has brought my family closer together”), acceptance (e.g., “has led me to be more accepting of things”), adjustment/coping (e.g., “has led me to cope better with stress and problems”), productivity/responsibility (e.g., “has made me a more productive [responsible] person”), gratefulness (e.g., “has made me more grateful for each day”), patience (e.g., “has taught me to control my temper”), and engagement in activities (“has renewed my interest in participating in different activities”). General instructions informed individuals to answer each item in relation to the “past 3 weeks” in regard to “coronavirus and its associated consequences.” Scale reliability for the averaged 14-items was high (α = 0.89).

#### Well-Being

Ten measures were included to assess individuals' emotional and psychological health. The purpose in selecting these scales is that they have been used extensively in past research on meaning (Zika and Chamberlain, 1992; Diener and Diener, 1995; Steger et al., 2006; Steger, 2018) and benefit finding (Cassidy et al., 2014; Slattery et al., 2017). For all measures, participants were asked to respond with how they felt over “the past 3 weeks” in the hope of capturing well-being as a function of government-mandated “stay at home” orders. Assessments included the: (a) 10-item International Positive and Negative Affect Short-Form (I-PANAS-SF; Karim et al., 2011; α's = 0.79–0.82); (b) Perceived Stress Scale 4 (PSS-4; Cohen and Williamson, 1988; e.g., “how often have you felt you were unable to control the important things in your life;” α = 0.70); (c) 10-item Center for Epidemiologic Studies Depression scale (CESD-10; Andresen et al., 2013; e.g., “everything you do is an effort;” α = 0.85); (d) Five-item Satisfaction With Life Scale (SWLS; Diener et al., 1985; e.g., “The conditions of my life are excellent;” α = 0.89); (e) Five-item “presence” subscale from the Meaning in Life Questionnaire (MLQ; Steger et al., 2006; “I understand life's meaning;” α = 0.87); (f) Ryan and Frederick (1997) 7-item subjective vitality measure (e.g., “I feel alive and vital;” α = 0.78); (g) Single-Item Self-Esteem Scale (Robins et al., 2001; i.e., “I have high self-esteem”); (h) Five-items taken from the General Self-Efficacy Scale (GSF; Schwarzer and Jerusalem, 1995; e.g., “I remain calm when facing difficulties because of my coping abilities;” α = 0.91); (i) 7 (i.e., non-filler) optimism items from the Revised Life Orientation Test (LOT-R; Scheier et al., 1994; e.g., “Overall, I expect more good things to happen to me than bad;” α = 0.73); and (j) 10-item Connor-Davidson Resilience Scale (CD-RISC 10; Campbell-Sills and Stein, 2007; e.g., “I am able to adapt to change;” α = 0.92). Responses to all measures were made on a 7-point scale ranging from 1 (*not at all*; *strongly disagree*) to 7 (v*ery true*; v*ery often*; s*trongly agree*).

Of the 10 outcome variables, three measures were not presented in their entirety (i.e., meaning in life, self-efficacy, and optimism). Our goal in doing this was to keep the study length short for compensation purposes (i.e., roughly ¼ the pay rate of minimum wage in the United States as recommended by MTurk). At the same time, the three excluded items on the optimism scale were filler and were not needed in the final computation of scores. The “search for meaning” subscale (Steger et al., 2006) was also excluded given its relationship with subjective well-being is debatable (Li et al., 2021). See Table 1 for descriptive statistics for the different measures.^4^ Preliminary analyses (i.e., correlations) between the variables of interest were also performed (see Table 2).

**Table 1:** Descriptive statistics for all scales (*N* = 238).

**Measure**	**Minimum**	**Maximum**	***M***	***SD***
FOC	1.00	7.00	4.16	1.44
FOD	1.00	7.00	4.27	1.55
BF	1.64	7.00	4.78	1.10
Positive affect (PA)	1.38	7.00	4.26	1.18
Negative affect (NA)	1.00	6.86	3.65	1.38
Stress	1.00	7.00	3.83	1.17
Depression	1.00	6.80	3.95	1.12
Life satisfaction (LS)	1.00	7.00	4.49	1.48
Meaning in life (MIL)	1.00	7.00	4.71	1.41
Vitality	1.00	7.00	4.37	1.34
Self-esteem (SE_1_)	1.00	7.00	4.55	1.61
Self-efficacy (SE_2_)	1.00	7.00	4.99	1.26
Optimism	1.00	5.00	3.08	0.88
Resilience	1.00	7.00	5.15	1.25

*Responses to all measures were made on a 7-point scale ranging from 1 (not at all; strongly disagree) to 7 (very true; very often; strongly agree)*.

**Table 2:** Preliminary analyses (i.e., correlations) between variables.

	**FOC**	**FOD**	**BF**	**PA**	**NA**	**Stress**	**Depression**	**LS**	**MIL**	**Vitality**	**SE_**1**_**	**SE_**2**_**	**Optimism**	**Resilience**
FOC	1.00													
FOD	0.439^**^	1.00												
BF	0.194^**^	0.190^**^	1.00											
PA	−0.125	−0.021	0.534^**^	1.00										
NA	0.301^**^	0.425^**^	−0.081	−0.335^**^	1.00									
Stress	0.187^**^	0.171^**^	−0.326^**^	−0.592^**^	0.578^**^	1.00								
Depression	0.232^**^	0.333^**^	−0.193^**^	−0.550^**^	0.707^**^	0.726^**^	1.00							
LS	0.052	0.052	0.433^**^	0.429^**^	−0.374^**^	−0.448^**^	−0.398^**^	1.00						
MIL	−0.029	−0.136^*^	0.493^**^	0.434^**^	−0.247^**^	−0.462^**^	−0.384^**^	0.591^**^	1.00					
Vitality	0.113	0.052	0.545^**^	0.624^**^	−0.291^**^	−0.513^**^	−0.418^**^	0.531^**^	0.485^**^	1.00				
SE_1_	−0.056	−0.098	0.425^**^	0.514^**^	−0.379^**^	−0.528^**^	−0.470^**^	0.524^**^	0.451^**^	0.469^**^	1.00			
SE_2_	−0.126	−0.070	0.466^**^	0.592^**^	−0.372^**^	−0.553^**^	−0.474^**^	0.549^**^	0.531^**^	0.478^**^	0.642^**^	1.00		
Optimism	0.059	−0.076	0.103	0.139^*^	−0.154^*^	−0.307^**^	−0.192^**^	0.121	0.090	0.332^**^	0.141^*^	0.115	1.00	
Resilience	−0.153^*^	−0.090	0.479^**^	0.599^**^	−0.366^**^	−0.579^**^	−0.475^**^	0.498^**^	0.465^**^	0.481^**^	0.565^**^	0.773^**^	0.134^*^	1.00

* *p ≤ 0.05*,

** *p ≤ 0.01*.

## Data Analysis Plan

The Statistical Package for the Social Sciences (SPSS Version 26) and MPlus Version 8.3 (Muthén and Muthén, 2017) were used to examine serial mediation (FOC→FOD→BF→well-being) through structural equation modeling (SEM). Assumption tests of normality were performed in SPSS by inspecting Shapiro-Wilk tests, skewness and kurtosis statistics, and histograms. Data were skewed for all measures except depression (Shapiro-Wilk tests: *p*s ≤ 0.033). Because of this, robust Maximum Likelihood Mean Adjusted (MLM) estimation was utilized in MPlus.

Given the non-normality of the data and large sample size (*N* = 238), model fit was assessed by using alternatives to the traditional chi-square test: specifically, the comparative fit index (CFI; Bentler, 1990); the robust root mean square error of approximation (RMSEA; Steiger, 1990); and the standardized root mean square residual (SRMR). Goodness of fit was defined as CFI ≥ 0.95, RMSEA < 0.05, and SRMR < 0.05; however, researchers have also argued adequate model fit with RMSEA values up to 0.08, CFI values > 0.90, and SRMR values up to 0.06 or 0.08 (e.g., Crombie et al., 2005; Lee et al., 2008; Kline, 2016). Modification indices from MPlus were used to improve structural fit by specifying covariances among indicators for the well-being latent variable. This thus made the direct paths between predictors high in face validity and theoretically meaningful. Each parameter added resulted in a statistically significant improvement in model fit. Modifications ended once a good model fit was obtained. The MODEL INDIRECT command was used to obtain standardized values and significance tests for indirect paths between variables (i.e., serial mediation).

## Results

A confirmatory factor analysis (CFA) on the proposed latent variable (i.e., positive well-being) was conducted prior to running the SEM analysis. The results revealed that life satisfaction, meaning presence, self-efficacy, resilience, and vitality were significant indicators. There were no significant findings for optimism, self-esteem, and positive and negative affect. To improve SEM model fit, modification indices were utilized to specify the co-varying relationships between life satisfaction and MIL and between self-efficacy and resilience. With these specifications, the latent variable demonstrated good model fit (CFI > 0.999; RMSEA ≤ 0.001 (90% CI: 0.001, 0.098), *p* = 0.728; SRMR = 0.009). The remainder of the SEM model was thus specified.

**Figure 1:**
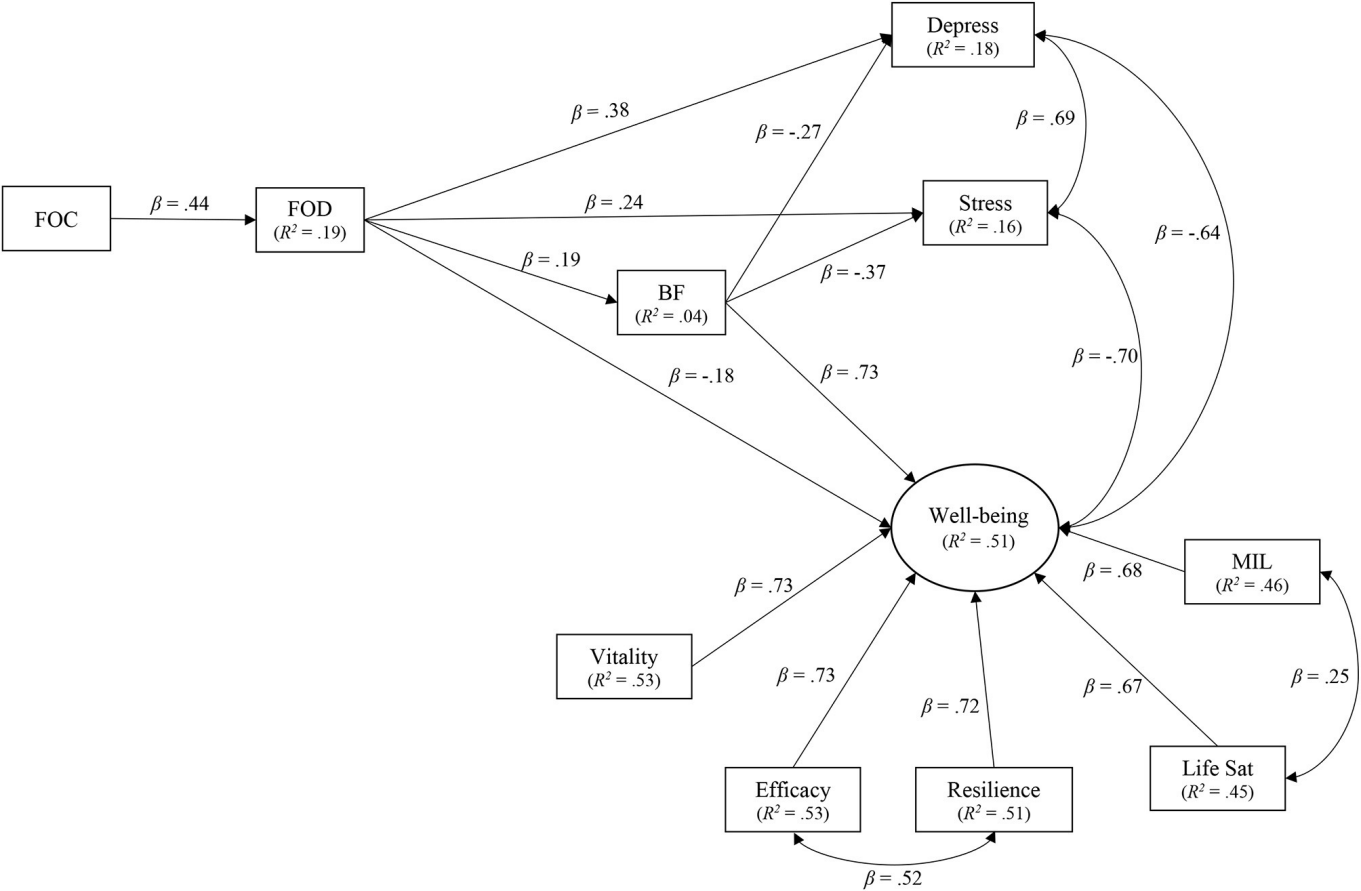
Final model. All paths (solid lines) shown are significant at *p* ≤ 0.010. FOC, fear of COVID-19; FOD, fear of death; BF, benefit finding; MIL, meaning in life.

The model was arranged such that FOC would predict FOD, which would predict BF. Benefit finding, in turn, would be related to positive well-being (i.e., latent variable), stress, and depression. Through modification indices, direct paths were also specified between FOD and the three outcome variables (i.e., well-being, stress, and depression). The final model demonstrated good fit: CFI = 0.966; RMSEA = 0.072 (90% CI: 0.047, 0.096), *p* = 0.068; SRMR = 0.046; WRMR = 0.896 (see Table 3 for inferential statistics and Figure 1 for a visual depiction of the model).^5^ The overall results indicated that higher FOC was positively associated with heightened death concerns (Hypothesis 1). Increased FOD, in turn, was related to greater feelings of depression, stress, and BF (Hypothesis 2) but lower well-being (see Table 4 for inferential statistics associated with the indirect effects). Of interest, higher BF in association with increased death concerns connected to coronavirus anxieties was predictive of heightened well-being, indicated by reduced feelings of depression and stress and higher psychological health (i.e., latent variable; Hypothesis 3).

**Table 3:** Standardized regression coefficients (β) and standard errors (*SE*) for direct paths.

**Effect**	**β**	***SE***	***p***
FOC→FOD	0.44	0.05	≤0.001
FOD→BF	0.19	0.07	0.010
FOD→Well-being	−0.18	0.06	0.004
FOD→Depression	0.38	0.05	≤0.001
FOD→Stress	0.24	0.06	≤0.001
BF→Well-being	0.73	0.06	≤0.001
BF→Depression	−0.23	0.06	≤0.001
BF→Stress	−0.37	0.07	≤0.001
Stress↔Well-being	−0.70	0.05	≤0.001
Depress↔Well-being	−0.64	0.05	≤0.001
Depress↔Stress	0.69	0.03	≤0.001
Well-being→MIL	0.68	0.05	≤0.001
Well-being→Self-efficacy	0.73	0.04	≤0.001
Well-being→Resilience	0.72	0.04	≤0.001
Well-being→Vitality	0.73	0.04	≤0.001
Well-being→Life satisfaction	0.67	0.05	≤0.001
Self-efficacy↔Resilience	0.52	0.05	≤0.001
Life satisfaction↔MIL	0.25	0.07	0.001

**Table 4:** Standardized regression coefficients (β) and standard errors (*SE*) for indirect effects.

**Indirect effect**	**β**	***SE***	***p***
FOC→FOD→BF	0.08	0.04	0.017
FOC→FOD→Well-being	−0.08	0.03	0.009
FOC→FOD→Depression	0.17	0.03	≤ 0.001
FOC→FOD→Stress	0.11	0.03	0.001
FOC→FOD→BF Well-being	0.06	0.03	0.019
FOC→FOD→BF→Depression	−0.02	0.01	0.043
FOC→FOD→BF→Stress	−0.03	0.02	0.034
FOD→BF→Well-being	0.14	0.06	0.012
FOD→BF→Depression	−0.05	0.02	0.032
FOD→BF→Stress	−0.07	0.03	0.023

## Discussion

Throughout the past year, we have seen unprecedented costs associated with the coronavirus pandemic. Not only is it physically threatening due to its contagiousness, symptom severity, and mortality association, but it has also led to declines in personal, social, economic, and political well-being in persons around the world. From the perspective of TMT (Pyszczynski et al., 2015), people can achieve psychological equanimity by validating their cultural beliefs, acquiring and/or maintaining feelings of self-worth, and pursuing and investing in close relationships. Unfortunately, right now, the world is inundated with existential anxieties both literally (e.g., news reports, COVID-19 trackers, social media) and symbolically via compromised worldviews, unemployment, social isolation from friends and family, and diminished emotional, physical, and psychological health. With the world becoming even more confusing and chaotic, it is becoming increasingly difficult to manage the terror of death.

Frankl (1959) and other growth-oriented theorists (e.g., Yalom, 1980; Deci and Ryan, 1985; Wong and Tomer, 2011; Martela and Steger, 2016), however, have argued that people have the potential to create a meaningful reality even amid heightened pain and suffering. The pursuit of life purpose is also evident during the COVID-19 pandemic (Sawyer and Brewster, 2019; De Jong et al., 2020; Trzebiński et al., 2020). Building on this, the current work explored the associative link between coronavirus and death worries and how they might be connected to stress-related growth and well-being. To test this idea, three hypotheses were examined. First, we expected a positive relationship between COVID concerns and fears of mortality. The first path of our SEM model supported this prediction in that a higher FOC was correlated with increased mortality fears. Not only were we able to replicate the results of past research examining the association between terror management processes and disease salience (e.g., Arrowood et al., 2017), but we were able to observe these events occurring at the time of an actual viral outbreak in the United States and the world.

Additionally, the search for life meaning is elemental to health, with some researchers suggesting it can result in post-traumatic growth instead of leading to stress (see e.g., Bower et al., 2009; De Jong et al., 2020). Higher levels of meaning in life, in turn, have been associated with reduced anxiety, distress (Sawyer and Brewster, 2019), and mortality awareness (Routledge and Juhl, 2010), even during COVID-19 (Trzebiński et al., 2020). Our second hypothesis thus examined the extent to which increased existential worries related to coronavirus concerns can contribute to greater meaning-making from BF. The second path of the mediational SEM model supported this hypothesis in that higher personal growth emerged for participants to the extent that death concerns were related to COVID worries. These findings are noteworthy as TMT has been criticized for its overemphasis on unconscious defensive drives and actions in the face of existential uncertainties (e.g., Wong and Tomer, 2011). Here, we were able to demonstrate, in real time during a deadly pandemic, that people can learn and grow from adversity through gratefulness, patience, appreciation, and by investing in themselves and others. Although researchers have only recently emphasized the positive trajectories of terror management (see Vail et al., 2012), this study is important as no TMT work, to our knowledge, has demonstrated post-traumatic growth via BF in response to heightened mortality awareness.

The final aim of this study was to explore carryover effects of increased BF on well-being. Prior research has shown that a meaningful life leads to higher mental and physical health (see e.g., Schippers and Ziegler, 2019 for a recent review). This is also true for persons who can flourish and grow (i.e., BF) when confronted with stressful experiences (e.g., illness, bereavement, war, terrorism; see e.g., Bower et al., 2009). The current work hypothesized that benefits from death-related coronavirus fears would be associated with higher emotional and psychological well-being. These findings were demonstrated in our overall SEM model with participants reporting higher collective health (e.g., vitality, self-efficacy, resilience, life satisfaction, and meaning presence) and reduced stress and depression along with the greater reported BF stemming from death and COVID fears. Consistent with the existential-humanistic tradition in which hope can help people cope with adversity, including death (e.g., Frankl, 1959; Yalom, 1980), the current research provides optimism: The positive effects (i.e., BF) stemming from stressful experiences (i.e., coronavirus pandemic, death salience) can lead to subjective happiness and better health.

Interestingly, our findings also showed that participants experienced lower well-being and higher feelings of depression and stress when BF was not included as a variable in the model (i.e., when only an associative link was made between FOC and FOD). These effects indicate that BF is one mechanism by which persons can develop intra- and interpersonal resources to promote adaptation in response to life stressors. Although beyond the scope of the present experiment, it would be valuable to understand this link more fully. For instance, does BF only emerge when people have their terror management needs met (e.g., close relationships, belief validation, self-esteem maintenance) following reminders of death? Additionally, if the current results are presumed to come from relatively healthy populations (e.g., college students), what happens to those who are psychologically overwhelmed by ineffective terror management defense mechanisms (e.g., PTSD; i.e., anxiety-buffer disruption theory [ABDT; Yetzer and Pyszczynski, 2019])? Future research should examine these possibilities.

The current work also has implications for both terror management and meaning-making literatures. First, it contributes to a broader understanding of TMT. Whereas reminders of death can (sometimes) function to generate negativity and defensiveness (the “dark side”), it too moves people toward positive life trajectories and beneficial outcomes (e.g., forgiveness, helping). Through death contemplation, individuals may put their life in a broader context to help achieve significance and purpose (e.g., Cozzolino et al., 2004). In a year during which many persons have been constantly threatened by potential illness and death (i.e., COVID-19), we argue that people can obtain benefits from the situation to flourish and be healthy (e.g., emotion, physical, psychological, social). As suggested by others (e.g., Deci and Ryan, 1985), there is some bidirectionality between reducing fear and living a meaningful and satisfying life; by achieving significance and purpose, individuals become aware that their life will continue to have meaning, even after death.

Second, much of the research on adversity and growth, even within the BF tradition, has focused extensively on personal trauma (e.g., bereavement, illness, violence; see e.g., Updegraff et al., 2008). It is important to understand how people can create meaning in everyday situations as they too can have implications for health and happiness. Adding to this, having life purpose is critical in terms of overcoming aversiveness (e.g., anxiety, depression, health problems) and promoting longevity (see e.g., Bower et al., 2009; Schippers and Ziegler, 2019; De Jong et al., 2020 for recent reviews). By understanding where people find meaning, and the positive carryover effects of such, interventions can be developed to improve mental and physical well-being. For instance, researchers are interested in the process of “life crafting” whereby persons reflect, set, and achieve goals (e.g., social, career, leisure) to create meaning and significance (see e.g., de Jong et al.). By means of expressive writing, people can rebuild meaning in life, and benefit from doing so, even if disrupted by grief from the COVID-19 pandemic (de Jong et al.). This is especially important for young adults, on which half our current sample was based (i.e., college students), as this population typically struggles with meaninglessness and increased mental health problems (see e.g., Schippers and Ziegler, 2019).

Although the present findings are encouraging, there are several limitations that warrant discussion. One concern has to do with sample size and the need to remove a significant number of participants (*n* = 213). As of late, researchers have raised concerns about fundamental shifts in MTurk data quality (see e.g., Chmielewski and Kucker, 2019). Chmielewski and Kucker (2019), for example, conducted a study in multiple waves over 4 years to find lower response quality in relation to data inconsistencies, improbable reactions, unusual comments, and in the use of bots and/or “farms” (i.e., specific servers utilized to bypass MTurk location restrictions). At the same time, researchers also believe it is possible to obtain high quality data from MTurk with extensive cleaning, with our study following all recommended strategies (Chmielewski and Kucker, 2019). The inclusion of MTurk participants helped increase the external validity of our findings, and further, the two samples only slightly differed only on depression and stress scores of all the measures used. Given our achieved power, however, future work should attempt to replicate our findings with a larger, more representative sample of participants.

An added concern has to do with the heterogeneity of our sample – that is, utilizing both college-aged and MTurk samples within the same study. As noted by others (Chandler et al., 2019), although more diverse, MTurk workers are non-representative of the population in the United States. They are mostly young, with 70% being below the age of 40. Mechanical Turk workers are also politically liberal, better educated, score lower on religiosity, are single, and without children as compared to the population at large (Chandler et al., 2019). Future research would benefit from replicating the current study in a sample of participants who closely resemble that of Americans (e.g., Prime Panels). At the same time, however, panel participants have been shown to fail screening questionnaires at a higher rate than MTurk individuals (Chandler et al., 2019). When they do pass, their performance is on par with Mechanical Turk workers.

Another limitation has to do with the correlational nature of the study design, along with the inclusion of a writing prompt for purposes of the larger study (see Swets and Cox, 2021). Supplementary analyses revealed no significant effect of the prime on any of our variables of interest (see Footnote 1). However, it is possible that the obtained results could be exaggerated by additional thoughts of COVID. Future research would benefit from (a) an experimental design whereby thoughts of mortality and coronavirus are manipulated to then measure personal growth and well-being (in a controlled laboratory setting); (b) by removing the influence of any extraneous variables; and (c) looking at the obtained effects longitudinally (i.e., true mediation) rather than one-time statistical associations (MacKinnon, 2012).

It would also be interesting to see if the current results hold considering cultural and demographic differences. Specifically, the present data was collected in the United States soon after COVID-19 was declared a worldwide pandemic (i.e., March, 11, 2020; World Health Organization [WHO]). At this time, our case numbers were still relatively low compared to other countries (i.e., Italy, Spain). The current findings may thus be exaggerated in nations where existential concerns are particularly high. It also seems possible that a positivity bias in BF and well-being may have been evident in Americans in April as the coronavirus threat was (comparatively) less salient. Now that the United States has the highest number of COVID-related cases and deaths [Centers for Disease Control Prevention (CDC), 2021], it would be interesting to see if the same pattern of results were to emerge later in 2020 or extending into later years.

Finally, the present study consisted predominantly of Caucasian persons (i.e., 69%), with very few minority participants. According to Centers for Disease Control Prevention (CDC) (2021) statistics, Black, Hispanic, and Native American individuals infected with coronavirus are about four times more likely to be hospitalized when compared to others and die at disproportionately higher rates than White persons. These disparities are explained, in part, to health care inequalities, along with social and economic factors (e.g., socioeconomic status, being essential workers, public transportation use). Not only can life meaning vary among persons with different cultural backgrounds (e.g., individualism vs. collectivism), but the ability to achieve meaningfulness and significance may be particularly challenging considering demographics and the stress of daily living.

Despite these limitations, this is the first study from a TMT perspective (to our knowledge) to examine predictors (i.e., COVID fears, mortality concerns) and mechanisms (i.e., BF) that contribute to the pursuit of meaning with carryover effects to well-being. These findings are encouraging because they show that personal growth is not specific to traumatic events (e.g., Bower et al., 2009), but rather, may stem from everyday experiences. At present, a heightened awareness of death and detriments in meaning (e.g., compromised beliefs) are affecting most individuals worldwide. For instance, although close relationships can serve as a basic form of comfort and security considering heightened fear, people have been forced to separate due to social distancing and safety precautions, with loved ones suffering and dying in isolation. By understanding the associative link between illness and mortality salience, and the possible meaning one can derive from existential vulnerabilities (e.g., growth, appreciation, BF), we can help people to better cope with COVID-19 and associated consequences. The degree to which individuals can understand such factors may restore a sense of security and hasten adaptive processes.

## Data Availability Statement

The raw data supporting the conclusions of this article will be made available by the authors, without undue reservation.

## Ethics Statement

The studies involving human participants were reviewed and approved by Texas Christian University Institutional Review Board. Written informed consent to participate in this study was provided by the participants, who needed to be 18 years or older to participate.

## Author Contributions

CC and JS conceived the original project idea and ran statistical analyses. Data was collected by CC, JS, and MY. CC wrote the manuscript with support from JS, BG, JX, and MY. All authors contributed to the article and approved the submitted version.

## Conflict of Interest

The authors declare that the research was conducted in the absence of any commercial or financial relationships that could be construed as a potential conflict of interest.

## Footnotes

^1^A power analysis was conducted for a RMSEA structural equation model (Preacher and Coffman, 2006; http://quantpsyc.org). With α = 0.05, *df* = 27, and power = 0.80, the recommended number of participants was 350. Although we exceeded this recruitment number, with the removal of cases due to bad data, a *post-hoc* power analysis was performed. Our achieved power was 0.58.

^2^As described in Swets and Cox (2021), a prime was introduced whereby participants thought about isolation considering COVID-19 or a daily event (i.e., control condition). Supplementary analyses showed no significant effect of the manipulation on the included variables of interest, *p*s ≥ 0.252.

^3^The creation of the FOC scale was based, in part, on Templer (1970) Death Anxiety Scale, the most widely used measure of mortality concerns (i.e., “The coronavirus holds nothing for me to fear” vs. “I feel the future hold nothing for me to fear”). In the time since data for this study was collected, researchers have developed a fear of COVID-19 scale (Ahorsu et al., 2020).

^4^The final SEM model found non-significant results for positive and negative affect, self-esteem, and optimism. As argued elsewhere (see e.g., Pyszczynski et al., 2015), this is consistent with hundreds of other TMT studies demonstrating no effect of heightened mortality salience on participants' affect scores. This has led researchers to argue that reminders of death produce a *potential* for anxiety (or negative affect), which is often averted with the activation of terror management defense mechanisms.

^5^The chi-square test detected poor model fit, χ^2^_(27)_ = 60.21, *p* < 0.001. Following others (Hu and Bentler, 1999; Ekas et al., 2010), the χ^2^*/df* ratio was utilized with a proportion of <5 being indicative of adequate fit. The model's χ^2^*/df* ratio was 2.23.

## References

[B1] Ahorsu D. K. Lin C. Y. Imani V. Saffari M. Griffiths M. D. Pakpour A. H. (2020). The fear of COVID-19 scale: development and initial validation. Int. J. Mental Health Addict. 1–9. 10.1007/s11469-020-00270-8. [Epub ahead of print]. PMC7100496 32226353

[B2] Andresen E. M. Byers K. Friary J. Kosloski K. Montgomery R. (2013). Performance of the 10-item center for epidemiologic studies depression scale for caregiving research. SAGE Open Med. 1:205031211351457. 10.1177/2050312113514576 26770693 PMC4687763

[B3] Arndt J. Cook A. Goldenberg J. L. Cox C. R. (2007). Cancer and the threat of death: the cognitive dynamics of death thought suppression and its impact on behavioral health intentions. J. Pers. Soc. Psychol. 92, 12–29. 10.1037/0022-3514.92.1.12 17201539

[B4] Arrowood R. B. Cox C. R. Kersten M. Routledge C. Shelton J. T. Hood R. W. (2017). Ebola salience, death-thought accessibility, and worldview defense: a terror management theory perspective. Death Stud. 9, 585–591. 10.1080/07481187.2017.1322644 28436743

[B5] Barrett F. S. Grimm K. J. Robins R. W. Wildschut T. Sedikides C. Janata P. (2010). Music-evoked nostalgia: affect, memory, and personality. Emotion 10, 390–403. 10.1037/a0019006 20515227

[B6] Becker E. (1973). The Denial of Death. New York, NY: Free Press.

[B7] Bentler P. M. (1990). Comparative fit indices in structural models. Psychol. Bull. 107, 238–246. 10.1037/0033-2909.107.2.238 2320703

[B8] Bower J. E. Moskowitz J. T. Epel E. (2009). Is benefit finding good for your health? Pathways linking positive life changes after stress and physical health outcomes. Curr. Direct. Psychol. Sci. 18, 337–341. 10.1111/j.1467-8721.2009.01663.x

[B9] Burke B. L. Martens A. Faucher E. H. (2010). Two decades of terror management theory: a meta-analysis of mortality salience research. Personal. Soc. Psychol. Rev. 14, 155–195. 10.1177/1088868309352321 20097885

[B10] Campbell-Sills L. Stein M. B. (2007). Psychometric analysis and refinement of the connor-davidson resilience scale (CD-RISC): validation of a 10-item measure of resilience. J. Trauma. Stress 20, 1019–1028. 10.1002/jts.20271 18157881

[B11] Carver C. S. Antoni M. H. (2004). Finding benefit in breast cancer during the year after diagnosis predicts better adjustment 5 to 8 years after diagnosis. Health Psychol. 23, 595–598. 10.1037/0278-6133.23.6.595 15546227

[B12] Cassidy T. McLaughlin M. Giles M. (2014). Benefit finding in response to general life stress: measurement and correlates. Health Psychol. Behav. Med. 2, 268–282. 10.1080/21642850.2014.889570 25750781 PMC4346032

[B13] Centers for Disease Control Prevention (CDC). (2021). CDC COVID Data Tracker: Maps, Charts, and Data Provided by the CDC. Available online at: https://covid.cdc.gov/covid-data-tracker/#cases_casesper100klast7days (accessed April 22, 2021).

[B14] Chandler J. Rosenzweig C. Moss A. J. Robinson J. Litman L. (2019). Online panels in social science research: Expanding sampling methods beyond mechanical turk. Behav. Res. Methods 51, 2022–2038. 10.3758/s13428-019-01273-7 31512174 PMC6797699

[B15] Chmielewski M. Kucker S. C. (2019). An MTurk crisis? Shifts in data quality and the impact on study results. Soc. Psychol. Personal. Sci. 11, 464–473. 10.1177/1948550619875149

[B16] Cohen S. Williamson G. (1988). Perceived stress in a probability sample of the United States, in Claremont Symposium on Applied Social Psychology. The Social Psychology of Health, eds Spacapam S. Oskamp S. (Thousand Oaks, CA: Sage Publications), 31–67.

[B17] Coplan R. J. Hipson W. E. Archbell K. A. Ooi L. L. Baldwin D. Bowker J. C. (2019). Seeking more solitude: conceptualization, assessment, and implications of aloneliness. Pers. Individ. Diff. 148, 17–26. 10.1016/j.paid.2019.05.020

[B18] Cox C. R. Reid-Arndt S. A. Arndt J. Moser R. P. (2012). Considering the unspoken: the role of death cognition in quality of life among women with and without breast cancer. J. Psychosoc. Oncol. 30, 128–139. 10.1080/07347332.2011.633980 22269079 PMC3295244

[B19] Cozzolino P. J. Staples A. D. Meyers L. S. Samboceti J. (2004). Greed, death, and values: from terror management to “transcendence management” theory. Pers. Soc. Psychol. Bull. 30, 278–292. 10.1177/0146167203260716 15030620

[B20] Crombie G. Sinclair N. Silverthorn N. Byrne B. M. DuBois D. L. Trinneer A. (2005). Predictors of young adolescents' math grades and course enrollment intentions: gender similarities and differences. Sex Roles J. Res. 52, 351–367. 10.1007/s11199-005-2678-1

[B21] De Jong E. M. Ziegler N. Schippers M. C. (2020). From shattered goals to meaning in life: life crafting in times of the COVID-19 pandemic. Front. Psychol. 11:577708. 10.3389/fpsyg.2020.577708 33178081 PMC7593511

[B22] Deci E. L. Ryan R. M. (1985). Intrinsic Motivation and Self-Determination in Human Behavior. New York, NY: Plenum. 10.1007/978-1-4899-2271-7

[B23] Diener E. Diener M. (1995). Cross-cultural correlates of life satisfaction and self-esteem. J. Personal. Soc. Psychol. 68:653–63. 10.1037/0022-3514.68.4.653 7738768

[B24] Diener E. Emmons R. A. Larsen R. J. Griffin S. (1985). The satisfaction with life scale. J. Pers. Assess. 49, 71–75. 10.1207/s15327752jpa4901_13 16367493

[B25] Ducharme J. (2020, May 27). COVID-19 has killed more than 100,000 Americans. Time. Available online at: https://time.com/5843349/coronavirus-death-toll-100000/ (accessed April 22, 2021).

[B26] Ekas N. V. Lickenbrock D. M. Whitman T. L. (2010). Optimism, social support, and well-being in mothers of children with autism spectrum disorder. J. Autism Dev. Disord. 40, 1274–1284. 10.1007/s10803-010-0986-y 20195734

[B27] Frankl V. E. (1959). Man's Search for Meaning: An Introduction to Logotherapy. Boston, MA: Beacon.

[B28] Fromm E. (1941). Escape From Freedom. New York, NY: Holt.

[B29] George L. S. Park C. L. (2014). Existential mattering: bringing attention to a neglected but central aspect of meaning?, in Meaning in Positive and Existential Psychology, eds Batthyany A. Russo-Netzer P. (New York, NY: Springer), 39–51.

[B30] Goldenberg J. L. Arndt J. (2008). The implications of death for health: a terror management health model for behavioral health promotion. Psychol. Rev. 115, 1032–1053. 10.1037/a0013326 18954213

[B31] Gosling S. D. Rentfrow P. J. Swann W. B. J. Jr. (2003). A very brief measure of the Big-five personality domains. J. Res. Pers. 37, 504–528. 10.1016/S0092-6566(03)00046-128810502

[B32] Hart J. Shaver P. R. Goldenberg J. L. (2005). Attachment, self-esteem, worldviews, and terror management: evidence for a tripartite security system. J. Personal. Soc. Psychol. 88, 999–1013. 10.1037/0022-3514.88.6.999 15982118

[B33] Heflick N. A. Goldenberg J. L. Keroack L. J. Cooper D. P. (2011). Grim reaping psychological well-being: repeated death contemplation, intrinsic motivation, and depression (Unpublished manuscript). Tampa, FL: University of South Florida.

[B34] Helgeson V. S. Reynolds K. A. Tomich P. L. (2006). A meta-analytic review of benefit finding and growth. J. Consult. Clin. Psychol. 74, 797–816. 10.1037/0022-006X.74.5.797 17032085

[B35] Hill P. L. Turiano N. A. (2014). Purpose in life as a predictor of mortality across adulthood. Psychol.Sci. 25, 1482–1486. 10.1177/0956797614531799 24815612 PMC4224996

[B36] Hu L. Bentler P. M. (1999). Cutoff criteria for fit indexes in covariance structure analysis: Conventional criteria versus new alternatives. Struct. Eq. Model. 6, 1–55. 10.1080/10705519909540118

[B37] Karim J. Weisz R. Rehman S. U. (2011). International positive and negative affect schedule short-form (I-PANAS-SF): Testing for factorial invariance across cultures. Proc. Soc. Behav. Sci. 15, 2016–2022. 10.1016/j.sbspro.2011.04.046

[B38] Kasser T. Sheldon K. M. (2000). Of wealth and death: materialism, mortality salience, and consumption behavior. Psychol. Sci. 11, 352–355. 10.1111/1467-9280.00269 11273398

[B39] Kline R. B. (2016). Principles and Practice of Structural Equation Modeling, 4th Edn. New York, NY: Guilford Press.

[B40] Kosloff S. Greenberg J. (2009). Pearls in the desert: death reminders provoke immediate derogation of extrinsic goals, but delayed inflation. J. Exp. Soc. Psychol. 45, 197–203. 10.1016/j.jesp.2008.08.022

[B41] Lee S. W. Stewart S. M. Byrne B. M. Wong J. P. S. Ho S. Y. Lee P. W. H.. (2008). Factor structure of the Center for epidemiological studies depression scale in Hong Kong adolescents. J. Personal. Assess. 90, 175–184. 10.1080/00223890701845385 18444112

[B42] Li J. B. Dou K. Liang Y. (2021). The relationship between presence of meaning, search for meaning, and subjective well-being: a three-level meta-analysis based on the meaning in life questionnaire. J. Happiness Stud. 22, 467–489. 10.1007/s10902-020-00230-y

[B43] Lifton R. J. (1979). The Broken Connection: On Death and Continuity of Life. New York, NY: Simon and Schuster.

[B44] Lykins E. L. B. Segerstrom S. C. Averill A. J. Evans D. R. Kemeny M. E. (2007). Goal shifts following reminders of mortality: Reconciling posttraumatic growth and terror management theory. Personal. Soc. Psychol. Bull. 33, 1088–1099. 10.1177/0146167207303015 17578931

[B45] MacKinnon D. P. (2012). Introduction to Statistical Mediation Analysis. New York, NY: Routledge. 10.4324/9780203809556

[B46] Martela F. Steger M. F. (2016). The three meanings of meaning in life: distinguishing coherence, purpose, and significance. J. Positive Psychol. 11, 531–545. 10.1080/17439760.2015.1137623

[B47] May R. (1953). Man's Search for Himself. New York, NY: Norton.

[B48] Mikulincer M. Florian V. Hirschberger G. (2003). The existential function of close relationships: introducing death into the science of love. Personal. Soc. Psychol. Rev. 7, 20–40. 10.1207/S15327957PSPR0701_2 12584055

[B49] Mosley A. J. Branscombe N. R. (2020). Benefit-finding improves well-being among women who have experienced gender discrimination. Sex Roles 84, 404–417. 10.1007/s11199-020-01175-5

[B50] Muthén L. K. Muthén B. O. (2017). Mplus User's Guide, 8th Edn. Los Angeles, CA: Muthén and Muthén.

[B51] Owens G. P. Steger M. F. Whitesell A. A. Herrera C. J. (2009). Posttraumatic stress disorder, guilt, depression, and meaning in life among military veterans. J. Trauma. Stress 22, 654–657. 10.1002/jts.20460 19924820

[B52] Park C. L. (2010). Making sense of the meaning literature: an integrative review of meaning making and its effects on adjustment to stressful life events. Psychol. Bull. 136, 257–304. 10.1037/a0018301 20192563

[B53] Preacher K. J. Coffman D. L. (2006). Computing power and minimum sample size for RMSEA [Computer software]. Available online at: http://quantpsy.org/ (accessed April 22, 2021).

[B54] Pyszczynski T. Abdollahi A. Solomon S. Greenberg J. Cohen F. Weise D. (2006). Mortality salience, martyrdom, and military might: the great satan versus the axis of evil. Personal. Soc. Psychol. Bull. 32, 525–537. 10.1177/0146167205282157 16513804

[B55] Pyszczynski T. Solomon S. Greenberg J. (2015). Thirty years of terror management theory: from genesis to revelation. Adv. Exp. Soc. Psychol. 52, 1–70. 10.1016/bs.aesp.2015.03.001

[B56] Rank O. (1936). Will Therapy and Truth and Reality. New York, NY: Knopf.

[B57] Reker G. T. Wong P. T. P. (1988). Aging as an individual process: toward a theory of personal meaning, in Emergent Theories of Aging, eds Birren J. E. Bengtson V. L. (Springer Publishing Company), 214–246.

[B58] Robins R. W. Hendin H. M. Trzesniewski K. H. (2001). Measuring global self-esteem: construct validation of a single-item measure and the rosenberg self-esteem scale. Pers. Soc. Psychol. Bull. 27, 151–161. 10.1177/0146167201272002

[B59] Rogers R. Sanders C. S. Vess M. (2019). The terror management of meaning and growth: How mortality salience affects growth-oriented processes and the meaningfulness of life, in Handbook of Terror Management Theory, eds (Elsevier Academic Press), 325–345. 10.1016/B978-0-12-811844-3.00014-7

[B60] Routledge C. Juhl J. (2010). When death thoughts lead to death fears: mortality salience increases death anxiety for individuals who lack meaning in life. Cogn. Emot. 24, 848–854. 10.1080/02699930902847144

[B61] Ryan R. M. Frederick C. M. (1997). On energy, personality and health: subjective vitality as a dynamic reflection of well-being. J. Personal. 65, 529–565. 10.1111/j.1467-6494.1997.tb00326.x 9327588

[B62] Sawyer J. S. Brewster M. E. (2019). Assessing posttraumatic growth, complicated grief, and psychological distress in bereaved atheists and believers. Death Stud. 43, 224–234. 10.1080/07481187.2018.1446061 29509067

[B63] Scheier M. F. Carver C. S. Bridges M. W. (1994). Distinguishing optimism from neuroticism (and trait anxiety, self-mastery, and self-esteem): a re-evaluation of the life orientation test. J. Pers. Soc. Psychol. 67, 1063–1078. 10.1037/0022-3514.67.6.1063 7815302

[B64] Schimel J. Simon L. Greenberg J. Pyszczynski T. Solomon S. Waxmonsky J.. (1999). Stereotypes and terror management: evidence that mortality salience enhances stereotypic thinking and preferences. J. Personal. Soc. Psychol. 77, 905–926. 10.1037/0022-3514.77.5.905 10573872

[B65] Schippers M. C. Ziegler N. (2019). Life crafting as a way to find purpose and meaning in life. Front. Psychol. 10:2778. 10.3389/fpsyg.2019.02778 31920827 PMC6923189

[B66] Schwarzer R. Jerusalem M. (1995). Generalized self-efficacy scale, in Measures in Health Psychology: A User's Portfolio. Causal and Control Beliefs, eds Weinman J. Wright S. Johnston M. (Windsor: Nfer-Nelson), 35–37.

[B67] Slattery É. McMahon J. Gallagher S. (2017). Optimism and benefit finding in parents of children with developmental disabilities: the role of positive reappraisal and social support. Res. Dev. Disabil. 65, 12–22. 10.1016/j.ridd.2017.04.006 28432893

[B68] Steger M. F. (2018). *Meaning and Well-Being*, in Handbook of Well-Being, eds Diener E. Oishi S. Tay L. (Salt Lake City, UT: DEF Publishers). Available online at: https://nobascholar.com (accessed April 22, 2021).

[B69] Steger M. F. Frazier P. Oishi S. Kaler M. (2006). The meaning in life questionnaire: assessing the presence of and search for meaning in life. J. Counsel. Psychol. 53, 80–93. 10.1037/0022-0167.53.1.80

[B70] Steiger J. H. (1990). Structural model evaluation and modification: an interval estimation approach. Multivariate Behav. Res. 25, 173–180. 10.1207/s15327906mbr2502_4 26794479

[B71] Steinman C. T. Updegraff J. A. (2015). Delay and death-thought accessibility: a meta-analysis. Personal. Soc. Psychol. Bull. 41, 1682–1696. 10.1177/0146167215607843 26443599

[B72] Swets J. A. Cox C. R. (2021). Singlehood, death concerns and benefit finding during the coronavirus (COVID-19) pandemic. Department of Psychology, Texas Christian University (Manuscript submitted for publication).

[B73] Taylor S. E. (1983). Adjustment to threatening events: a theory of cognitive adaptation. Am. Psychol. 38, 1161–1173. 10.1037/0003-066X.38.11.11617971297

[B74] Templer D. I. (1970). The construction and validation of a death anxiety scale. J. Gen. Psychol. 82, 165–177. 10.1080/00221309.1970.9920634 4394812

[B75] Tomich P. L. Helgeson V. S. (2004). Is finding something good is the bad always good? Benefit finding among women with breast cancer. Health Psychol. 23, 16–23. 10.1037/0278-6133.23.1.16 14756599

[B76] Trzebiński J. Cabański M. Czarnecka J. Z. (2020). Reaction to the COVID-19 pandemic: the influence of meaning in life, life satisfaction, and assumptions on world orderliness and positivity. J. Loss Trauma 25, 544–577. 10.1080/15325024.2020.1765098

[B77] Updegraff J. A. Silver R. C. Holman E. A. (2008). Searching for and finding meaning in collective trauma: results from a national longitudinal study of the 9/11 terrorist attacks. J. Pers. Soc. Psychol. 95, 709–722. 10.1037/0022-3514.95.3.709 18729704 PMC2617710

[B78] Vail K. E. III. Juhl J. Arndt J. Vess M. Routledge C. Rutjens B. T. (2012). When death is good for life: considering the positive trajectories of terror management. Personal. Soc. Psychol. Rev. 16, 303–329. 10.1177/1088868312440046 22490977

[B79] Vail K. E. III. Rothschild Z. K. Weise D. R. Solomon S. Pyszczynski T. Greenberg J. (2009). A terror management analysis of the psychological functions of religion. Personal. Soc. Psychol. Rev. 14, 84–94. 10.1177/1088868309351165 19940284

[B80] Wong P. T. P. Tomer A. (2011). Beyond terror and denial: the positive psychology of death acceptance. Death Stud. 35, 99–106. 10.1080/07481187.2011.535377 24501830

[B81] Yalom I. (1980). Existential Psychotherapy. New York, NY: Basic Books.

[B82] Yetzer A. M. Pyszczynski T. (2019). Terror management theory and psychological disorder: ineffective anxiety-buffer functioning as a transdiagnostic vulnerability factor for psychopathology, in Handbook of Terror Management Theory, ed Routledge C. Vess M. (Cambridge, MA: Academic Press), 417–47. 10.1016/B978-0-12-811844-3.00018-4

[B83] Zika S. Chamberlain K. (1987). Relation of hassles and personality to subjective well-being. J. Pers. Soc. Psychol. 53, 155–162. 10.1037/0022-3514.53.1.155

[B84] Zika S. Chamberlain K. (1992). On the relation between meaning in life and psychological well-being. Br. J. Psychol. 83, 133–145. 10.1111/j.2044-8295.1992.tb02429.x 1559144

